# Controls of Soil Spatial Variability in a Dry Tropical Forest

**DOI:** 10.1371/journal.pone.0153212

**Published:** 2016-04-21

**Authors:** Sandeep Pulla, Jean Riotte, H. S. Suresh, H. S. Dattaraja, Raman Sukumar

**Affiliations:** 1 Centre for Ecological Sciences, Indian Institute of Science, Bangalore, 560012, India; 2 Indo-French Cell for Water Sciences, Indian Institute of Science, Bangalore, 560012, India; 3 Géosciences Environnement Toulouse (IRD-Université Paul Sabatier-CNRS), 31400, Toulouse, France; Tennessee State University, UNITED STATES

## Abstract

We examined the roles of lithology, topography, vegetation and fire in generating local-scale (<1 km^2^) soil spatial variability in a seasonally dry tropical forest (SDTF) in southern India. For this, we mapped soil (available nutrients, Al, total C, pH, moisture and texture in the top 10cm), rock outcrops, topography, all native woody plants ≥1 cm diameter at breast height (DBH), and spatial variation in fire frequency (times burnt during the 17 years preceding soil sampling) in a permanent 50-ha plot. Unlike classic catenas, lower elevation soils had lesser moisture, plant-available Ca, Cu, Mn, Mg, Zn, B, clay and total C. The distribution of plant-available Ca, Cu, Mn and Mg appeared to largely be determined by the whole-rock chemical composition differences between amphibolites and hornblende-biotite gneisses. Amphibolites were associated with summit positions, while gneisses dominated lower elevations, an observation that concurs with other studies in the region which suggest that hillslope-scale topography has been shaped by differential weathering of lithologies. Neither NO_3_^−^-N nor NH_4_^+^-N was explained by the basal area of trees belonging to Fabaceae, a family associated with N-fixing species, and no long-term effects of fire on soil parameters were detected. Local-scale lithological variation is an important first-order control over soil variability at the hillslope scale in this SDTF, by both direct influence on nutrient stocks and indirect influence via control of local relief.

## Introduction

Soil heterogeneity is thought to play a key role in the origin and maintenance of tropical plant diversity and community structure at regional [1,2], landscape [3,4] and local (<1 km^2^) [5,6] scales. At the local scale, this heterogeneity may promote coexistence of plant species by niche segregation [7] and help maintain spatially-structured genetic variation [8]. Similarly, soil resource variability and heterogeneity are linked to community structure and diversity of soil biota [9]. In addition, an understanding of local-scale soil variability can inform ecosystem restoration design, such as during the selection of appropriate vegetation for a site [10]. Despite these implications, little is understood about the patterns and controls of spatial variability in tropical forest soils.

At the local scale, climate being constant, soil variability broadly arises from variation in parent material, topography and organismal activity [11], with additional factors such as fire being important in some regions [12]. These factors influence soil formation at different spatial and temporal scales and, while it is generally accepted that they act in a hierarchical fashion, their relative dominance may vary by biome [13]. Our focus here is on seasonally dry tropical forests (SDTFs), a globally extensive biome that is characterized by several dry months (precipitation < 100mm) [14]. Soil-forming factors that dominate in SDTFs may thus be potentially different from those that dominate in aseasonal wet forests and grass-dominated savannas. SDTFs are relatively understudied despite being the most widespread of tropical forests [15]. Below, we briefly review studies from SDTFs that relate to the roles of four potentially important controls—lithology, topography, vegetation and fire—on local-scale soil variability.

### Lithology

Studies from other forest types show that the effect of lithological variation on soils can be significant at local or smaller scales (e.g. [16]). SDTF soil studies tend to assume spatially uniform parent material influence at the local scale (e.g. [17,18]), although studies at landscape or larger scales do incorporate parent material variation [19]. To our knowledge, no SDTF study has examined the relationship between lithology and soil properties at the local scale.

### Topography

The relationship between local-scale topography and soil properties in SDTFs is relatively well studied, largely focusing variation across broad topographic categories (e.g. ridge vs. valley, north- vs. south-facing slopes) (e.g. [17,20–22,18]). Topography is recognized as an important local-scale control in SDTFs, though soil variation does not always appear to follow the classic catena pattern wherein lower elevations tend to have the higher soil moisture, nutrient status and clay content. The role of topographic aspect in affecting soil properties by influencing solar radiation interception has also been documented in SDTFs [21].

### Vegetation

Tree species-soil associations have been studied in SDTFs [6], but it is generally unclear to what extent these associations are driven by habitat requirements of tree species versus their impact on soils. In this context, the frequently-observed relationship between N-fixing trees and soil N (e.g. [23–25]), which is likely to have resulted from the effects of plants on soils, as opposed to the other way around, merits study in SDTFs.

### Fire

Studies from other forests suggest that the impact of fire on soils includes numerous short- and long-term changes to soil chemistry, physical properties and biological activity [26,27]. Patchy fires can potentially affect local-scale soil variability both indirectly, by modifying plant-soil interactions [28], and directly, by modifying soil properties in burnt patches. Although many SDTFs burn regularly [29], the effect of fire on SDTF soils appears to largely have been studied in context of land-use change [30,31]; few studies in a natural context exist [28].

Our objectives are to assess the roles of lithology, topography, vegetation and fire as controls of soil spatial variability in a SDTF in southern India. Specifically, we hypothesized the following:

- Soil properties vary with variation in lithology at the local scale.
- Soil properties vary systematically along hillslopes in accordance with the catena concept. Further, soil properties differ significantly on southern and northern aspects.
- Soil N is greater under N-fixing tree species belonging to the family Fabaceae [32].
- Soil properties change with increasing fire frequency (defined here as the number of times a patch was burnt during the study period) due to accumulation of multiple long-term changes.

## Materials and Methods

### Study site

The study was conducted in a permanent 50-ha plot (the Mudumalai Forest Dynamics Plot) located in the transition zone between tropical moist- and dry-deciduous forest in Mudumalai Wildlife Sanctuary (MWLS), Tamil Nadu, southern India (11° 35′ N 76° 32′ E, 910–1030 m above sea level, 1000 m E-W x 500 m N-S). The site has been continuously monitored since 1988 [33]. Mean annual precipitation at Kargudi, located ~ 4 km from the plot, during 1989–2013 was 1245 ± 278 mm. Mean monthly maximum and minimum temperatures at Kargudi during 1990–2013 were 27.4°C and 16.4°C, respectively. Permissions to conduct research in Mudumalai were obtained from the Tamil Nadu Forest Department.

MWLS lies in the Moyar-Bhavani shear zone of the Western Dharwar Craton which separates two 2.5 Ga-old charnockite massifs. Although dominated by Achaean gneissic granitoids (“peninsular gneiss” [34]), the shear zone exhibits a variety of lithologies, such as amphibolite facies (e.g. hornblendite, pyroxenite) produced by retrograde metamorphism [35,36] and some late intrusions of granite, pegmatite and quartzite dykes or veins crossing the gneiss foliation [37]. The initially thick, lateritic regolith cover set up on the Deccan Plateau during the Mesozoic was denudated during several Cenozoic erosion cycles [38], an important consequence of which was rejuvenation of the saprolite. The latter is immature, meaning it still contains all primary bedrock minerals except biotite [39]. Though less abundant, most of the primary minerals, such as Na-plagioclase, sericite, hornblende and quartz, are recovered in the soils developed on such saprolites, as observed in a watershed in Mule Hole (Survey of India: Mūlehole), located in a similar geological, climatic and vegetation context about 17-km NW of the plot [39–41]. Soils in the study region have been classified as Udic Haplustalfs (Alfisols) and Udic Argiustolls (Mollisols) with deep A horizons and high clay content [42].

The canopy in the study site is dominated by *Lagerstroemia microcarpa* Wt., *Terminalia crenulata* Roth., *Anogeissus latifolia* (DC.) Bedd. and *Tectona grandis* L. f. The understorey is characterized by *Kydia calycina* Roxb., *Helicteres isora* L., *Cassia fistula* L., *Catunaregam spinosa* (Thunb) Tirveng., *Phyllanthus emblica* L., and the alien invasive species *Lantana camara* L. and *Chromolaena odorata* (L.) King & Robinson. The forest floor is dominated by perennial tall grasses, mainly *Themeda cymbaria* Hac. and *Cymbopogon flexuosus* (Steudel) Watson. Logging of select species in the region in which the plot is located extended from at least the early part of the nineteenth century to 1968 [33]. The region also harbors a high density of large mammals that make substantial impacts on the vegetation through feeding and on soil through dung deposition. Although a management practice of fire-suppression is followed, dry-season, human-induced ground fires occur with an average fire-return interval of 6 years in the dry-deciduous forest [43].

### Sampling

Soil sampling for characterizing plant-available nutrients, Al, moisture and texture was performed towards the end of the first annual wet season (summer or southwest monsoon) in September-October 2004 following Centre for Tropical Forest Science (CTFS) soil-sampling protocols [5]. For soil chemistry analysis, 200 samples were collected on a 50-m resolution regular grid overlaid on the FDP with an additional 100 samples collected at distances of 2, 8 or 20 m in random directions from every alternate grid point. Samples were collected from the top 0–10 cm, excluding the O horizon. Available cations, B and P were extracted using the Mehlich-III extractant and analyzed using atomic emission inductively coupled plasma (AE-ICP) spectroscopy. N was extracted as NO_3_^−^-N and NH_4_^+^-N using 2.0 M KCl and measured colorimetrically using an auto analyzer (OI FS 3000, OI Analytical, Texas, USA). For soil physical analysis, the plot was gridded into 20x20 m cells and samples collected at the center of each cell (N = 1250). These samples were analyzed for gravimetric soil moisture, texture by feel (USDA, http://www.nrcs.usda.gov/wps/portal/nrcs/detail/soils/edu/?cid=nrcs142p2_054311) and Munsell color. Physical property values from the nearest 20x20 m cell were assigned to each of the 300 soil chemistry samples for analysis.

A smaller set (103) of grid locations was re-sampled in order to characterize total C during the dry season of December 2015 –January 2016. Samples were oven dried at 45–55°C, ground, and analyzed using a TOC analyzer (Shimadzu TOC-5000, Shimadzu Corporation, Kyoto, Japan).

In order to map potential parent material distribution, rock samples were collected opportunistically—aiming for an even spatial coverage—from numerous rocky outcrops occurring within and near the plot during June 2014 –March 2015. All woody plant species ≥1 cm in diameter at a height of 1.3m from the ground (diameter at breast height, DBH) within the plot were identified to species, measured for DBH, tagged, and mapped to the closest 0.5m during May 1988-May 1989 [44] following CTFS protocols [45]. Subsequent annual censuses recorded recruitment and mortality, while DBH was measured every fourth year starting 1988. The plot had 25,554 individuals of 72 species in 1988 and 15,533 individuals of 72 species in 2004. Quadrat burnt/unburnt status was recorded annually during 1988–2004 at the end of each dry season on a 10-m resolution grid. The plot was almost completely burnt in 1989, 1991, 1992, 1996, 2002 and 2010, and partially burnt in 1994, 1995 and 2004.

### Data preprocessing

The plot was gridded into 10x10-m quadrats (N = 5000) for subsequent analyses. A catchment-scale (4.5x4.5 km) digital elevation model (DEM) was obtained from stereo-pair satellite imagery (National Remote Sensing Centre, http://www.nrsc.gov.in) centered on the plot. Topographic variables derived from elevation were its first and second derivatives (i.e. slope inclination, slope aspect and curvature), a hydrological index of relative wetness (topographic wetness index (TWI) [46]) and insolation (annual global radiation [47]). TWI is formulated to represent a catenary position [48], and has been used to predict soil properties at local (e.g. [49]) and larger scales (e.g. [50]). All derived variable rasters were computed on the catchment-scale DEM from which the plot extent was subsequently extracted. This allowed the region within the plot to be affected by calculations on the region outside it, which is especially important for hydrological variables. TWI was calculated using SAGA 2.0.5 (System for Automated Geoscientific Analyses, http://www.saga-gis.org; SAGA Wetness Index module, default options). Slope inclination and aspect, curvature and insolation were calculated using ArcGIS 9.2 (ESRI, California, USA; Spatial Analyst Tools, default options). All further data processing and analyses were performed in R 3.0 [51]. Seven soil variable outliers were removed (S1 Appendix) and values below the detection limit of the AE-ICP were set to the machine detection limit for the corresponding element.

### Data analyses

To test if major outcrop lithologies were associated with specific topographic positions, pair-wise differences in the mean elevations of major lithologies were assessed for statistical significance using a restricted randomization test, i.e. lithologies were randomly assigned to elevations without changing their relative positions. This was achieved by toroidal transformations, wherein the elevation map was randomly translated and mirrored 5000 times following toroidal edge-wrapping rules and recalculating the test statistic—the difference in mean elevation for each pair of lithologies—for each such randomization in order to generate its null distribution [52].

**Fig 1:**
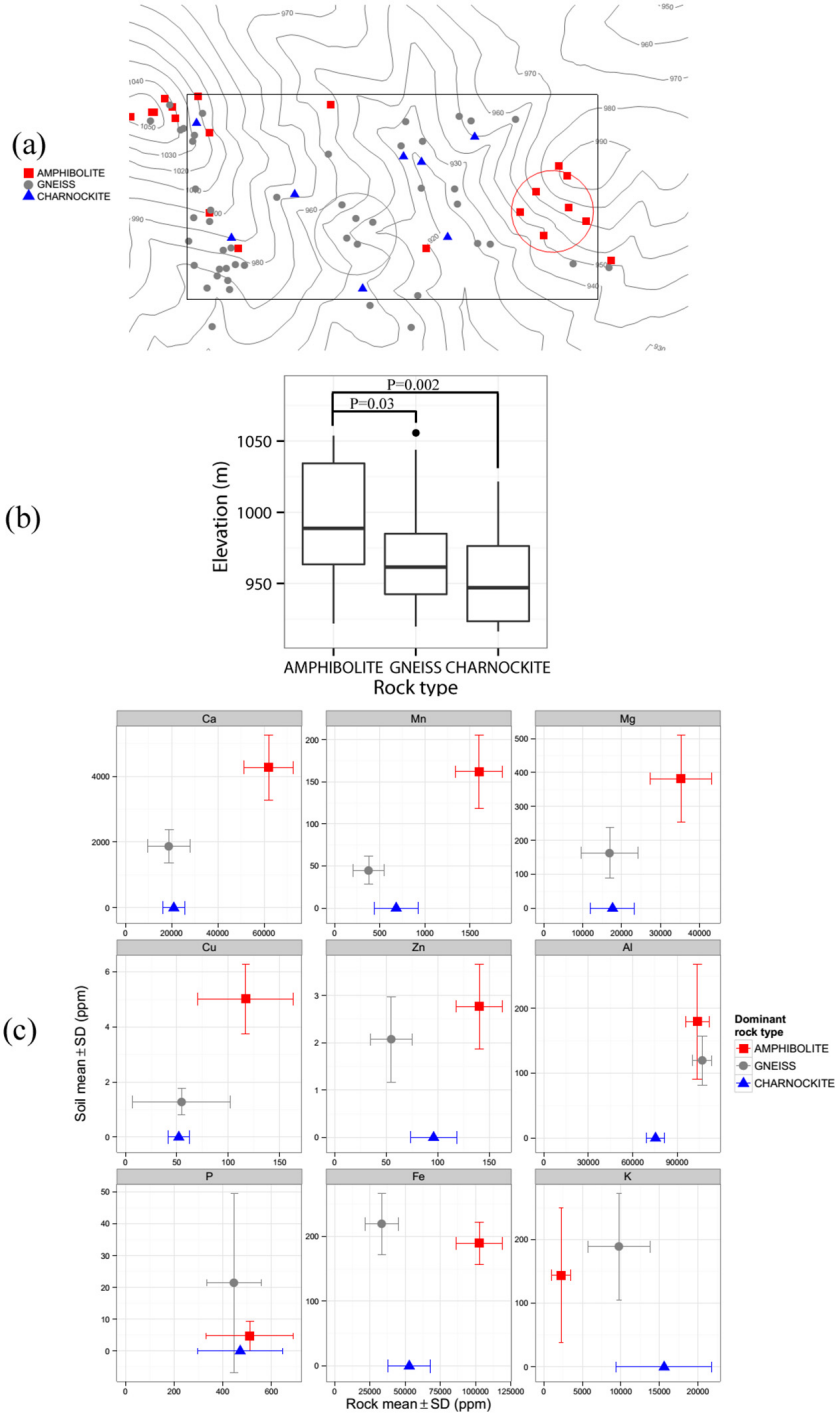
Comparison of rock outcrops and soil chemistry in the 50-ha plot. (a) Rock outcrop composition showing only amphibolites, gneisses and charnockites. Amphibolite- and gneiss-outcrop dominated patches are outlined by red and gray circles, respectively. The rectangle represents the 50-ha plot boundary; contours are 10 m; overlapping points have been jittered. (b) Boxplots showing elevation by rock type. *P*-values are from restricted randomization tests. (c) Graphs comparing rock composition (mean ± 1 SD) from nearby Mule Hole [39] with soil composition (mean ± 1 SD) from amphibolite- (n = 18) and gneiss- (n = 19) outcrop dominated patches shown in (a). Mule Hole data for gneiss is from boreholes BH1, BH5 and BH12 (n = 54), and for amphibolite from borehole BH6 (n = 24) [39]. Charnockite rock composition data (n = 5; no corresponding soil data; shown at the bottom of each graph) is from Samuel et al. [53]. See S2 Appendix for the complete outcrop map.

The outcrop survey suggested that the two dominant lithologies in the plot were amphibolites and gneisses. To test if the whole-rock geochemistry of these lithologies was associated with soil chemistry, the mean and standard deviation data of amphibolite- and gneiss- bedrock composition from Mule Hole [39] were compared with soil composition from two locations within the Mudumalai plot that were dominated by amphibolites and gneisses, respectively, as shown in Fig 1a.

Generalized least squares linear regression models were fit with soil variables as responses and topographic (elevation, slope, aspect, curvature, TWI, insolation) and vegetation (basal area of Fabaceae trees), and fire (times burnt during 1988–2004) variables as predictors. The full tree censuses immediately preceding each soil sampling event was used to calculate basal areas (2004 census in case of 2004 soil sampling; 2012 census in case of 2015–16 soil sampling). Because slope aspect is a circular variable, it was decomposed into two linear variables using the sine and cosine of aspect, representing “eastness” and “northness”, respectively. Because individual trees are known to affect soils up to 15-m away [24], basal areas were smoothed using a 15-m bandwidth isotropic Gaussian kernel to avoid edge effects (i.e. influences of adjacent trees outside a quadrat on soils within a quadrat); thus the basal area at any point was a weighted average of basal areas of nearby trees. Soil texture and pH were also included as predictors because, even though texture and pH are themselves affected by soil-forming factors, they potentially are stronger proximal controls of other soil properties. Texture determines water- and nutrient-holding capacities while several biogeochemical processes involved in nutrient cycling are pH dependent [12,54]. Because a rainfall event modified soil moisture conditions about halfway through sampling, we only used soil moisture data prior to the event. All predictors were centered and scaled by 1 standard deviation (SD) to avoid correlations with higher-order terms, improve numerical stability and make effect sizes of predictors comparable. Response variables were also centered and scaled to make effect sizes comparable across models.

## Results

**Fig 2:**
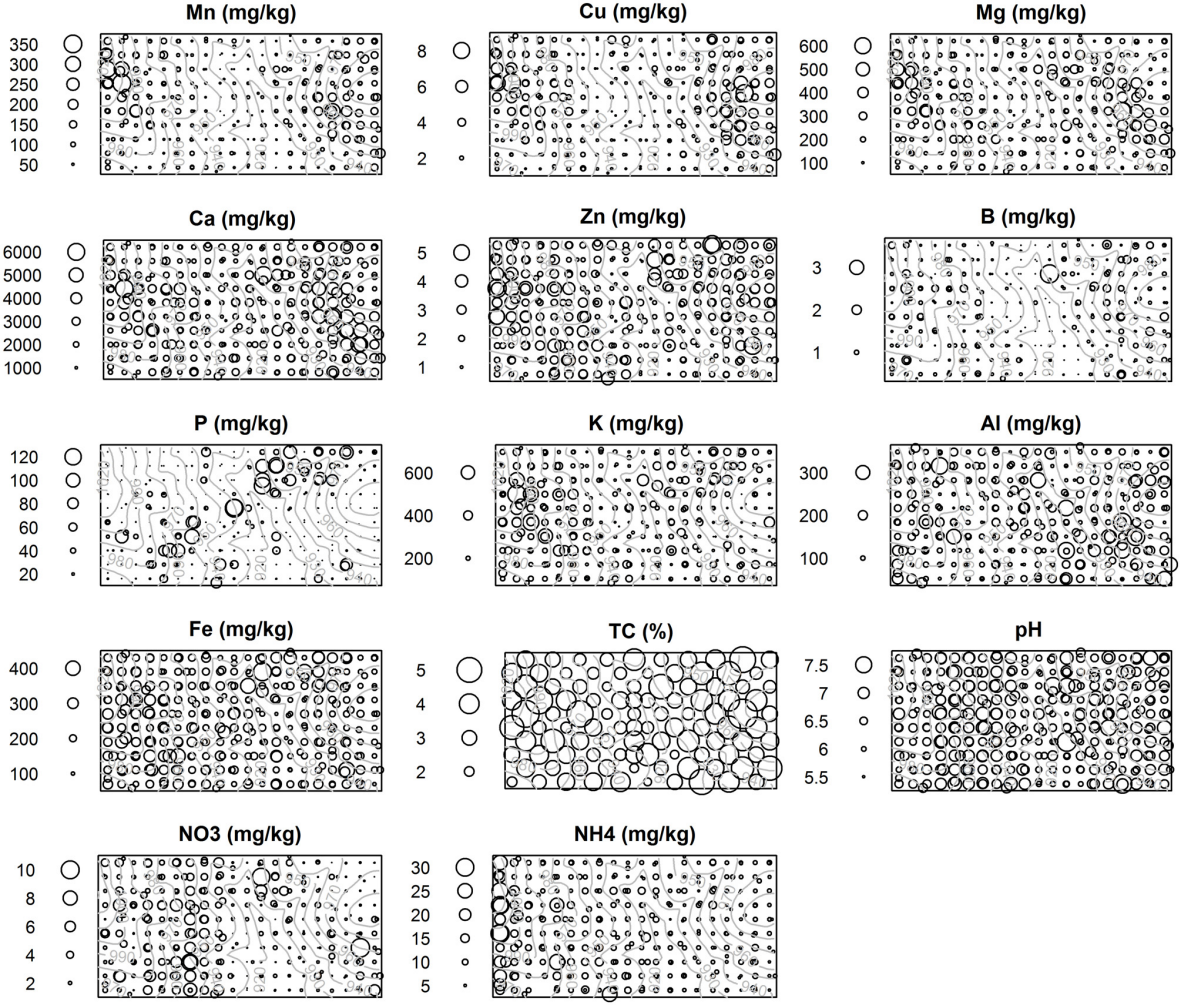
Spatial maps of soil properties sampled at 300 points in the 50-ha plot. TC = total carbon; NO3 = NO_3_^−^-N; NH4 = NH_4_^+^-N.

**Fig 3:**
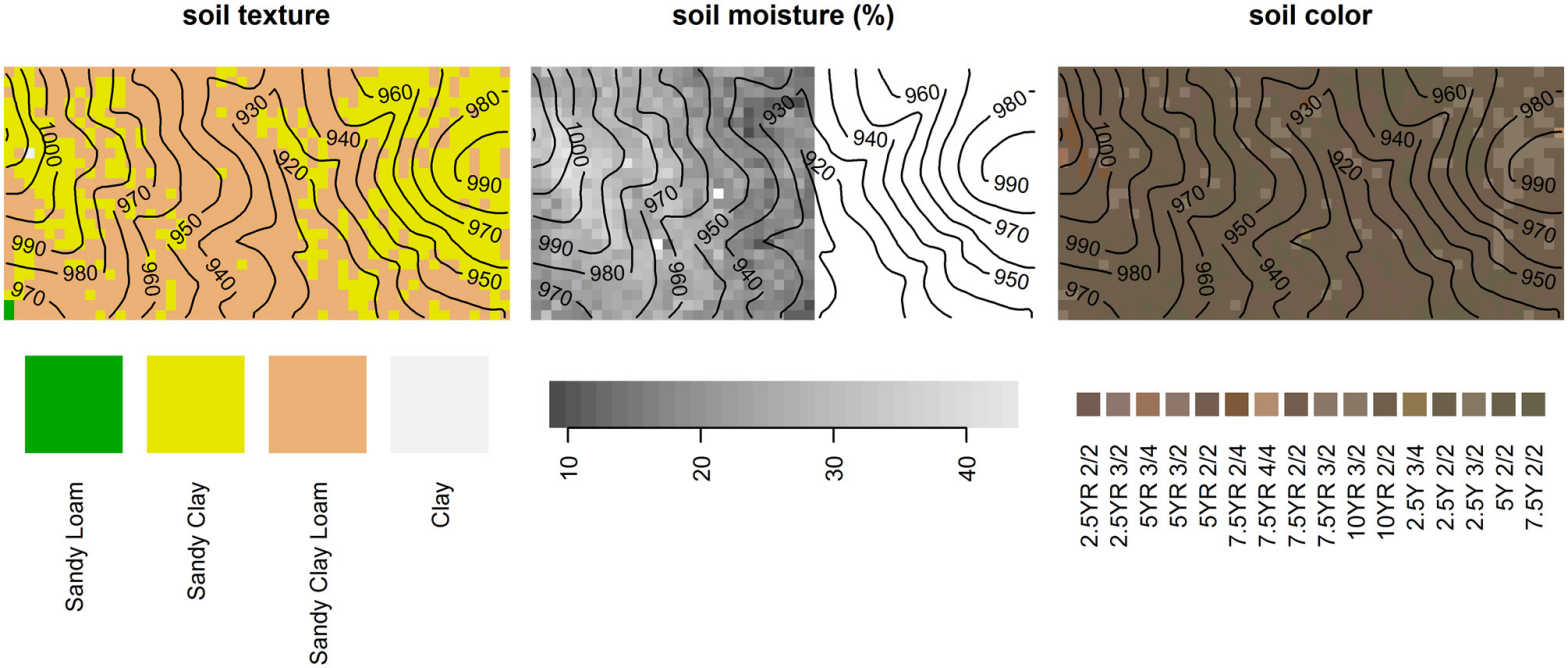
Spatial maps of soil properties sampled at 1250 points (centres of 20x20 m grid cells) in the 50-ha plot. A rainfall event modified soil moisture conditions during sampling; hence only soil moisture data prior to the event (left part of the plot) are shown.

**Fig 4:**
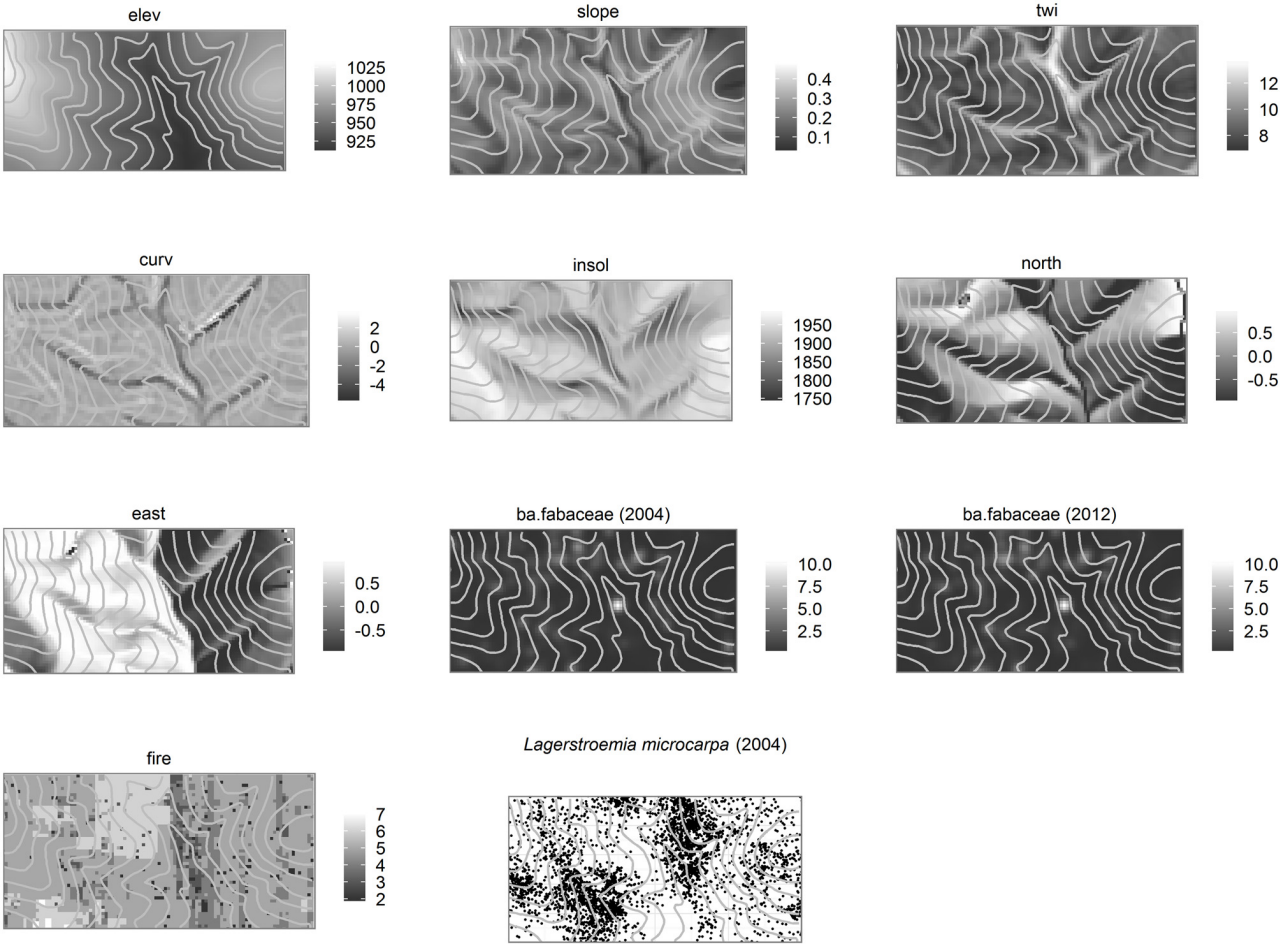
Spatial maps of topographic, vegetation and fire variables in the 50-ha plot. elev = elevation; slope = slope inclination; east = slope aspect (“eastness”); north = slope aspect (“northness”); curv = curvature; insol = insolation; twi = topographic wetness index; ba.fabaceae = basal area of woody Fabaceae species; fire = fire frequency (times burnt during 1988–2004). Elevation is in m; slope is in radians; insolation is in kWhm^-2^; fire is in 17 y^-1^.

Soil variable measurements are summarized in Table 1. P had the highest coefficient of variation across the plot while pH varied the least. Most plant-available nutrients, total carbon (TC) and pH were positively correlated with each other and negatively correlated with Al (Table 2). Spatial maps of soil and rock-outcrop sampling, and topographic, vegetation and fire variables are shown in Figs 2–4.

**Table 1:** Summary of soil variable measurements from the 50-ha plot.

Variable	Mean	SD	CV
pH	6.68	0.35	5.28
TC	3.47	0.91	26.21
moisture	23.93	6.66	27.84
Fe	215.58	62.42	28.95
Ca	2509.40	971.90	38.73
Zn	2.34	0.98	41.92
Al	137.88	64.14	46.52
Mg	205.71	101.21	49.20
K	230.11	120.57	52.39
NH_4_^+^-N	8.69	4.58	52.64
Cu	2.47	1.46	59.20
Mn	84.26	53.62	63.63
NO_3_^−^-N	2.63	1.75	66.68
B	0.65	0.45	69.33
P	20.21	27.45	135.84

SD = standard deviation; CV = coefficient of variation (%). All variables except pH, moisture and total carbon (TC) are expressed in mg kg^-1^. Moisture and TC are expressed in %. Variables are ordered by increasing CV. Outliers have been removed as described in S1 Appendix.

**Table 2:** Correlations (Pearson’s r) between soil variable measurements from the 50-ha plot.

	B	Mg	Al	P	K	Ca	Mn	Fe	Cu	Zn	NO_3_^−^-N	NH_4_^+^-N	pH
Mg	0.44***												
Al	-0.29***	0.04											
P	0.15	0.06	-0.25**										
K	0.49***	0.35***	-0.17*	0.41***									
Ca	0.67***	0.79***	-0.06	0.16	0.36***								
Mn	0.52**	0.65***	0.22	-0.23	0.25**	0.63***							
Fe	0.05	0.11	-0.03	0.58***	0.44***	0.11	-0.13						
Cu	0.41*	0.7***	0.19	-0.22	0.17	0.65***	0.8***	0					
Zn	0.5***	0.47***	-0.23**	0.43***	0.54***	0.49***	0.44**	0.38***	0.44**				
NO_3_^−^-N	0.3*	0.16	-0.31***	0.17	0.23**	0.11	0	0.25**	-0.04	0.26*			
NH_4_^+^-N	0.22**	0.24***	-0.09	0.02	0.3**	0.09	0.24***	0.2	0.18	0.4***	0.24		
pH	0.42***	0.07	-0.46***	0.15	0.14	0.24**	0.04	-0.09	-0.03	0.18	0.31***	-0.06	
TC	0.33**	0.5***	-0.07	0.02	0.23	0.55***	0.38**	0.09	0.52***	0.26*	-0.02	0.13	0.07

Statistically significant correlations are shown in **boldface**;

*P < 0.05,

**P < 0.01,

***P < 0.001 (*P*-values have been adjusted to take into account the spatially-autocorrelated nature of data [55] and for multiple hypothesis testing [56]).

Rock outcrops largely consisted of hornblende-biotite gneisses and amphibolites, with a smaller number of charnockites distributed in the plot. Amphibolite outcrops occurred at significantly higher elevations compared to gneisses (elevation difference 28.5 m, *P* = 0.03; toroidal randomization test) and charnockites (elevation difference 42 m, *P* = 0.002), whereas the mean elevations of gneisses and charnockites did not differ significantly (elevation difference 13.5 m, *P* = 0.23 N.S.) (Fig 1b). A comparison of amphibolite and gneiss bedrock composition from Mule Hole [39] and amphibolite and gneiss-outcrop dominated soils from the study plot is shown in Fig 1c and Table 3. Regional charnockite composition data from Samuel et al. [53] is also shown for reference in Fig 1c although no charnockite-outcrop dominated patches could be identified for comparison of soil chemistry. Ca, Mn, Mg, Cu and Zn were higher both in amphibolite bedrocks in Mule Hole and amphibolite-outcrop dominated soils in the plot, compared to gneiss bedrocks and gneiss-outcrop dominated soils, respectively. Al and P were similar in both bedrocks and soils. Fe was much higher in amphibolite bedrocks compared to gneiss but similar in soil samples. Similarly, K was higher in gneiss bedrocks compared to amphibolite but similar in soils.

**Table 3:** Comparison of bedrock composition [39] and plant-available ions in soils.

	Amphibolite bedrocks (n = 24)	Gneiss bedrocks (n = 54)	*Bedrocks amphibolite*: *gneiss ratio*	Amphibolite-outcrop dominated soils (n = 18)	Gneiss-outcrop dominated soils (n = 19)	*Soils amphibolite*: *gneiss ratio*
Ca	61989±10763	18837±9195	*3*.*3*: *1*	4271±991	1875±510	*2*.*3*: *1*
Mn	1600±260	377±175	*4*.*2*: *1*	162±44	45±17	*3*.*6*: *1*
Mg	35219±7918	16926±7292	*2*.*1*: *1*	382±129	163±75	*2*.*3*: *1*
Cu	117±47	55±47	*2*.*1*: *1*	5.02±1.27	1.29±0.48	*3*.*9*: *1*
Zn	141±22	55±20	*2*.*6*: *1*	2.77±0.9	2.07±0.9	*1*.*3*: *1*
Al	103700±7862	106912±6486	*1*: *1*	180±89	120±38	*1*.*5*: *1*
P	511±179	446±112	*1*.*1*: *1*	4.76±4.62	21.45±28.22	*1*: *4*.*5*
Fe	102700±16556	33565±11764	*3*.*1*: *1*	189±33	219±48	*1*: *1*.*2*
K	2257±1198	9766±4033	*1*: *4*.*3*	144±106	189±84	*1*: *1*.*3*

All values are mean ± 1 SD in mg kg^-1^.

**Fig 5:**
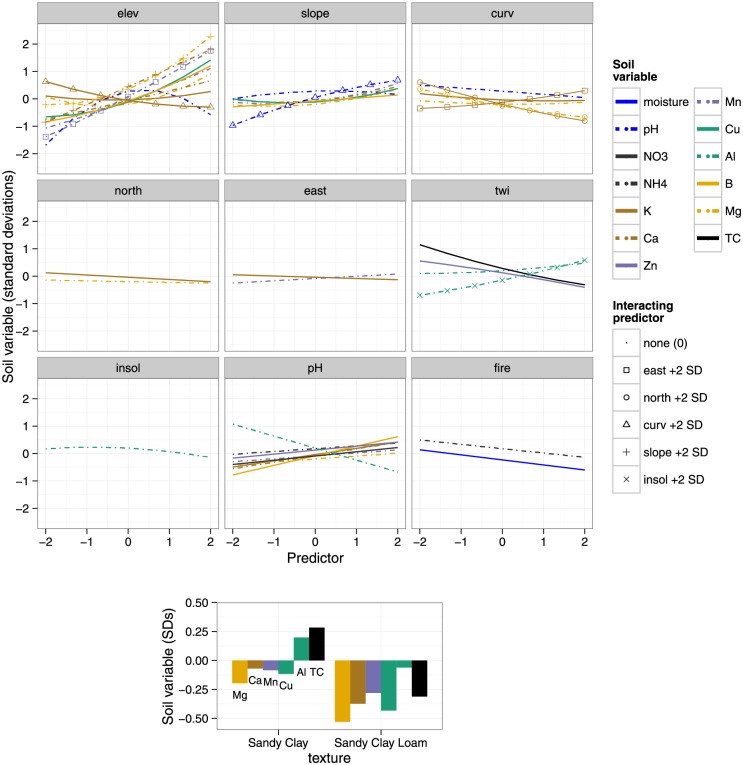
Predictions for statistically-significant terms in soil variable regressions. No predictors were significant for soil P and Fe. elev = elevation; slope = slope inclination; east = slope aspect (“eastness”); north = slope aspect (“northness”); curv = curvature; twi = topographic wetness index; insol = insolation; fire = fire frequency (times burnt during 1988–2004), TC = total carbon. All response variables and predictors except fire were standardized as described in the text; fire was centered but not scaled. Thus, the units for all variables are standard deviations (Tables 1 and 4), except fire, which is in 17 y^-1^. Predictions with interactions are represented by overlaid points; the legend indicates the point shape and interacting predictor value used in prediction (when only the main effect is shown, there are no overlaid points and interacting predictor values are 0). Not shown: distances x and y (see S3 Appendix for all regression terms).

Soil variable regression results are shown in Fig 5 (see also S3 Appendix); statistically significant coefficients, excluding small effect sizes (coefficient > 0.2, i.e. 1 SD change in the predictor results in > 0.2 SD change in the soil variable), are interpreted as follows. Frequently significant predictors included pH, elevation, texture and the x-coordinate. Elevation was frequently significant and had large effect sizes; each SD increase in elevation (= 26 m, Table 4) resulted in up to 0.54 SD change in the respective soil variable value (in decreasing order of effect size: Mn, Cu, B, Ca, pH, Mg, K).The quantities of all soil variables increased with elevation and slope, but pH peaked at intermediate elevations. Total C and Zn decreased with TWI. The significance, and occasionally large effect, of the x coordinate on some soil properties (S3 Appendix), implied the presence of large-scale east-west gradients that are not accounted for by any other topographic, vegetation, fire or soil variable included in the regressions.

**Table 4:** Summary of topographic and vegetation predictor variables used in soil variable regressions.

Variable	Mean	SD
Elevation (m)	958.27	25.97
Slope inclination (radians)	0.21	0.06
TWI	8.51	1.07
Curvature	0.00	0.83
Insolation (kWm^-2^)	1911.96	40.02
Slope aspect “eastness”	0.14	0.75
Slope aspect “northness”	-0.24	0.60
Smoothed basal area of Fabaceae trees in 2004	0.63	0.62
Smoothed basal area of Fabaceae trees in 2012	0.57	0.58

SD = standard deviation; All variable values are for 10x10-m quadrats.

Sandy clay soils tended to be found at higher elevations (mean = 968 m) than sandy clay loam soils (mean = 952 m), but the difference (16 m) was not significant by a toroidal randomization test (*P* = 0.26 N.S.). TC, Mg, Cu, Ca, Al and Mn were significantly lower in sandy clay loam soils compared to sandy clay soils. Higher pH was associated with higher nutrient availability and lower Al.

## Discussion

Bedrock composition in the Mudumalai plot was not uniform, with one of the clearer patterns being significantly greater amphibolite presence at the top of plot topography and the widespread occurrence of hornblende-biotite gneisses at lower elevations (Fig 1a and 1b; S2 Appendix). This configuration concurs with the one found in the neighboring Mule Hole watershed [57], located ~17-km NW of the Mudumalai plot. Both sites are located in the Moyar-Bhavani shear zone and exhibit considerable lithological heterogeneity at the local (<1 km^2^) scale, share the 1200-1300mm isohyet, and are covered by intact tropical dry deciduous forest. This allows for direct comparisons and for inferences to be carried over across sites. In Mule Hole, fresh amphibolite is found just below the soil layer, indicating that the saprolite hardly develops on this amphibolite lithology in the present climatic regime, in contrast to gneiss where biotite hydrolysis and greater prevalence of discontinuities [58] allows faster weathering and the development of a thick saprolite layer [39,57]. On the basis of observations in the Mudumalai plot and geological studies in Mule Hole, we suggest that topography at both sites has been shaped by differential weathering of lithologies, as is also widely observed at the landscape scale in the region [59,60]. According to Gunnell [38], denudation patterns in the Deccan Plateau regolith during the Cenozoic were shaped by the weatherability of rocks, allowing weathering-resistant lithologies such as massive charnockites and granites to emerge in the landscape against a background of peninsular gneisses.

Our results highlight several controls of soil spatial variability from a larger hierarchy [11,13] that dominate at the local scale in this SDTF. As discussed below, in terms of the magnitude of regression coefficients (Fig 5 and S3 Appendix), lithology appears to be most important, followed by soil texture and topography.

The first section below discusses elements whose spatial distributions in the plot are correlated with lithological variation. The subsequent section deals with elements that are immobile under present conditions. Next, elements that are not well explained by any of the considered factors are discussed. Finally, soil texture, moisture and pH patterns are discussed. We conclude by placing results from our study site in the larger geological and climatic context.

### Plant-available Ca, Mn, Mg, Cu, Zn and B, and total C

Regional amphibolite contains greater Ca, Mn, Mg, Cu and Zn, on average, as compared to gneiss [39,53,61], a pattern that is reflected in soil samples collected from amphibolite- and gneiss-outcrop dominated patches within the plot (Fig 1). Because elevation lacks an intrinsic meaning at the spatial scale of the plot, in the regressions, it either represents lithological variation, which is correlated with elevation (Fig 1b), or an unknown variable that happens to be correlated with both lithological variation and elevation. In the latter case, elevation could represent drainage or depth to the water table, although these properties typically are better captured by TWI, which was surprisingly not significant in most regressions. Indeed, the fundamental role that topography plays in soil development is embodied in the concept of a catena, referring to a series of soils along a slope that vary predictably as a result of debris and moisture flux and varying depth to the water table [62]. The observations in the Mudumalai 50-ha plot were the inverse of classic catena pattern wherein lower elevations are typically enriched in ions and organic matter due to inputs from higher elevations. Such inverse patterns have been reported from other SDTFs and have usually been attributed to removal of material from lower elevations in open catchment conditions [17,22,63]. Topography also affects incident solar radiation [21], which is highest on southern aspects in the northern hemisphere. However, regression coefficients of aspect and insolation were small, suggesting their limited influence at the scale of the plot.

We suggest that the presence of significantly greater Ca, Cu, Mn and Mg at higher elevations in the Mudumalai plot, representing differences of a factor of 2.3–3.9, is best explained by whole-rock geochemical differences of parent amphibolite compared to gneiss, that are also similarly large in magnitude (a factor of 2.1–4.2) (Table 3 and Fig 5). B appears to follow a similar spatial pattern as Ca, Mn, Mg and Cu, suggesting that B distribution may also be partly lithology-driven, although this is a conjecture as whole-rock B data is lacking. An alternate explanation for the observed B pattern is leaching in the drainage area. Zn shows a similar, but less clear pattern (Fig 2), which may be partly due to high Zn content in other lithologies such as charnockites (96±22 mg kg^-1^), outcrops of which infrequently occurred throughout the plot (Fig 1). Alternatively, like B, Zn depletion near stream channels (1 SD increase in TWI was associated with 0.24 SD decrease in Zn) may have resulted from leaching in the drainage area. Like Zn, TC decreases (-0.37 SD) with TWI, thereby increasing with elevation since TWI and elevation are negatively correlated. However, the variation in TC is strongly linked to soil texture, on average being 0.6 SD lower in sandy clay loam soils compared higher-elevation sandy clay soils. Such a positive correlation between carbon, organic matter and clay content is often observed (e.g. [17]), and is associated with the ability of clays to bind and protect organic matter from degradation. TC was also positively correlated with Ca, Mg, Mn, Cu, Zn and B (Table 2) indicating that the availability of these nutrients is also linked to texture and organic matter content.

### Plant-available Al and Fe

Regional amphibolite contains significantly greater Fe (10.27±1.66%) than does gneiss (3.36±1.18%), whereas there is no such contrast in Al (amphibolite: 10.37±0.79%; gneiss: 10.69±0.65%). Soil Fe and Al were similar in amphibolite- (Fe: 189±33; Al: 180±89; mg kg^-1^) and gneiss-outcrop (Fe: 219±48; Al: 120±38 mg kg^-1^) dominated patches. Low mobility of Fe in current sub-humid conditions may explain why, contrary to what is observed for base cations, the three-fold variation in bedrock Fe is not reflected in soil Fe, the latter also being poorly explained by topographic, vegetation and fire variables in the regressions. Dissolution of Fe takes place by protonation, reduction and complexation [64]. Studies from neighboring Mule Hole suggest that water tables in the region remain several meters deep [65,66], although seasonal perched water tables do form in valley-bottom black soils during the monsoon, resulting in reducing conditions in portions of the watershed [67]. If similar conditions exist at our study site, reducing conditions probably do not occur for the bulk of the year and in most parts in the plot. In addition, concentrations of dissolved organic carbon (DOC) in soil pore waters in Mule Hole are relatively low [68]. Fe is not expected to be mobile under these conditions of close to neutral pH (6.68±0.35), well-drained soils and low DOC. The solubility of Al would be similarly limited under these pH and DOC conditions, and its spatial variability within the plot was largely controlled by pH (1 SD increase in pH was associated with 0.43 SD decrease in Al) and soil texture (Fig 5).

### Plant-available N, P and K

None of these nutrients are significantly correlated with TC, indicating that their availability in soil is not linked with organic matter content of the soil. Because bedrock N was not characterized, soil N variation in the plot arising from variation in lithologies [69] is unknown. Symbiotic N-fixing tree species are known to increase soil N in their vicinity (e.g. [25]) through decomposition of N-rich litter [70]. Global data from the tropics show that N-fixing tree species in general [71], and Fabaceae (a family known to have several N fixing species) in particular [72], have greater foliar N compared to other tree species. However, basal area of trees belonging to the family Fabaceae was not significantly associated with higher content of either form of mineral N. Apparently N inputs from these trees do not dominate other sources of soil N at the scale considered, although the lack of a relationship may also be due to nitrate leaching in the drainage area (Fig 2). Another potential confounding factor is the observed W-E gradient in mineral N, particularly NH_4_^+^-N, which may have resulted from changing soil N stocks during our sampling—studies in northern Indian SDTFs have shown that N-mineralization and nitrification rates peak during the monsoon (mineralization rates of up to 48.5 mg kg^-1^ month^-1^ and nitrification rates of up to 28.3 mg kg^-1^ month^-1^ have been reported, compared to mean NH_4_^+^-N of 8.73 mg kg^-1^ and NO_3_^−^-N of 2.64 mg kg^-1^ in our study plot), during which time plant uptake is also maximized through proliferation of fine roots [20,73,74].

Amphibolite P content (0.05±0.02%) was similar to that of gneiss (0.04±0.01%), and soil P in patches dominated by either rock type did not appear to differ significantly (amphibolite: 4.76±4.62; gneiss: 21.45±28.22; mg kg^-1^). However, soil samples in the plot show distinct SW-NE trending P-rich patches (Fig 2). Such a pattern can potentially result from variation in bedrock lithology not revealed by the present outcrop survey, such as the presence of pegmatitic veins that are known to occur in the region [37]. The high stem density of a dominant canopy tree, *Lagerstroemia microcarpa* Wt. (Fig 4), in these P-rich patches suggests an alternate explanation for this pattern, namely that soils under *L*. *microcarpa* canopies could be enriched by P-rich litterfall. Although we did not test for soil-tree associations at a species level in the present study, the latter explanation seems unlikely considering the fact that (a) *L*. *microcarpa* ≥1 cm DBH were absent from some P-rich patches throughout the 17-year study period, and (b) the range of leaf litter P across tree species from southern Indian moist deciduous forests, which included *L*. *microcarpa* and six other species that occur at our study site [75], is far lesser (0.034–0.077%, a factor of ~2) than the observed variation in soil P in the plot (0.0001–0.0131%, a factor of ~112). Moreover, the range of foliar P of tropical tree species reported in global reviews does not exceed a factor of ~20 [71,72].

Charnockite samples from the region had the highest K (1.56±0.62%), followed by gneisses (0.98±0.4%) and amphibolites (0.23±0.12%) (Fig 1). However, the bulk of K in regional gneisses comes from biotite and sericite (57 and 41% of K_2_O in gneiss samples, respectively) [39], of which the former is rapidly lost at the weathering front level, thereby reducing the contrast in K across soils developed from gneisses and amphibolites. Additional soil K contrast is potentially introduced by the presence of charnockites. The factors affecting the spatial pattern of soil K in the plot remain unclear, although nutrient cycling by vegetation may play an important role considering the rapid uptake and high mobility of K in plants [54].

### Texture, moisture and pH

Soil texture was significant in regressions for TC, Mg, Cu, Ca, Al and Mn. The observed variation in texture may itself be linked to variation in parent material, particularly the two-fold greater Mg content in amphibolite lithology (Fig 1), which would favor smectite formation and vertic properties of soils [57].

Despite the presence of sandy clay texture in the central, low-elevation region of the plot (Fig 3), there is an absence of obvious clay-rich accretion zones at lower elevations. Such zones are expected to result from downslope fluxes of water and debris that cause nutrients and finer particles to accumulate at toeslopes and in depressions over time [12,76–78]. Lack of clay accretion zones in surficial soils at lower elevations could be explained via several processes: movement of clay down the profile, clay decomposition due to leaching out of Fe in alternating redox cycles [18]–particularly at the end of the rainy season, as has been observed in Mule Hole [67], and the open-catchment condition. The presence of reducing conditions in some regions at the edges of the stream channels is suggested by predominatly yellowish hues (2.5Y, 5Y) and low chroma (2) (Fig 3) [12].

Soil moisture variation was most likely driven by the greater water-holding capacity of sandy clay compared to sandy clay loam (Fig 3), although soil texture was not statistically significant in the moisture regression possibly due to the smaller sample size. The fire variable we used (times burnt during 1988–2004) was significantly associated with moisture: each additional year a quadrat was burnt was associated with a decrease in soil moisture by 0.18 SD (Fig 5). This association appears to be a spurious and is strongly influenced by the 2004 fire that was largely confined to a patch northwest of the valley, which also happens to have lower soil moisture at the surface due to the prevalence of sandy clay loam. Although widely observed in forest-fire studies, it seems unlikely that the decrease in soil moisture with frequent burning is related to fire-induced water repellency in soil, which is caused by vaporization and downward movement of hydrophobic substances [79]. During fires, soil temperatures 1 cm below the surface rarely reach 175–200°C in these forests [80], the range within which water repellency is maximized, and the effect of low severity fires on water repellency is typically short-lived [79].

Soil pH peaked at intermediate elevations and, despite its limited variation in the plot, was also associated the availability of B, K, Ca, Zn, Mg, Mn and both forms of mineral N (Fig 5).

### Geological and climatic context

Soils in our study site are less acidic, have much greater Ca and K content, and much lesser Al content compared to values reported from forest plots in the wetter tropics in Panama, Ecuador and Malaysia that used identical soil sampling protocols [5] (S4 Appendix). Contrary to wet tropical environments where all primary minerals but quartz are leached out of the soil and in arid environments where soil development is minimal, the sub-humid conditions prevailing in the present study site and neighboring Mule Hole watershed favor the presence of a significant fraction of cation-bearing minerals such as Na-plagioclase, smectite, chlorite and epidote in the soil layer [40,81]. For example, the large variation in bedrock Ca (amphibolite: 6.2±1.08%; gneiss: 1.88±0.92%) is reflected in soil Ca differences (amphibolite: Ca: 4271±991; gneiss: 1875±510, mg kg^-1^), suggesting that most of soil Ca variation is controlled by bedrock. High evapotranspiration rate in regional SDTFs [65,66,82] induces rapid oversaturation of the soil solution, limiting chemical weathering [83–85]. Wet-season nutrient loss due to leaching may be further reduced in SDTFs due to immobilization in microbes [86] as well as retention by plants [87]. Intense leaching of base cations in wetter forests should therefore result in a much weaker bedrock signature on *in situ* soils, although bedrock influence on vegetation can potentially still manifest at sites with deep- rooting plants or sites experiencing tectonically-driven erosion or fluvial erosion [88–90]. Rainfall and parent material may also determine the strength of topographic signature on soil heterogeneity: Khomo et al. [91] studied southern African granitic catenas in arid, semi-arid and sub-humid zones and concluded that heterogeneity of clay and base cations in catena soils peaked in the 400–800 mm annual rainfall range. In drier sites, distinct catena elements did not emerge, whereas in wetter sites, the bulk of the hillslope was highly leached, with only a narrow clay-accumulation zone occurring at toeslopes. The authors suggested that catenas underlain by more mafic or more felsic parent materials may differ in their mode of development. Topography undoubtedly plays an important role—albeit secondary to parent material, given the smaller regression coefficients—in generating soil spatial variability in this SDTF, as attested by the significance of topographic variables including slope inclination, aspect and curvature in the soil regressions.

Three factors appear to have ensured that the signature of bedrock heterogeneity is reflected in soils, by both direct influence on nutrient stocks and indirect influence via control of local relief. First, the stripping of lateritic regolith cover during the Cenozoic, as well as ongoing, periodic tectonic uplift [58] resulted in the rejuvenation of relatively immature, primary-mineral rich saprolite. Second, these relatively large nutrient stocks have been protected from intense leaching, compared to wetter tropics, as a result of present sub-humid conditions. Third, differential weathering of intermingling lithologies has been partially responsible for generation local-scale relief that is correlated with lithological variation and acts as a further control on soil spatial variability.

## Supporting Information

S1 Appendix List of soil variable outliers. (DOCX)

S2 Appendix Complete rock outcrop map of the 50-ha plot. (TIFF)

S3 Appendix Significant terms in soil variable regressions. No predictors were significant for P and Fe. elev = elevation; slope = slope inclination; east = slope aspect (“eastness”); north = slope aspect (“northness”); curv = curvature; insol = insolation; twi = topographic wetness index; ba.fabaceae = basal area of woody Fabaceae species; texture = soil texture class; fire = fire frequency (times burnt during 1988–2004); x, y = distances in west-east and south-north directions, respectively; TC = total carbon. Quadratic term of a given variable *var* is shown as *var*^2. All response variables and continuous predictors except fire were standardized as described in the text; fire was centered but not scaled. Quadratic and first-order interaction terms were included where deemed necessary, based on scatter plots and prior expectations. Predictors were tested for multicollinearity based on variance inflation factors (VIF) and correlations between coefficient estimates. Because TWI and insolation were correlated with the remaining topographic variables, separate regressions were built using two subsets of mutually uncorrelated predictors. Residual plots and empirical variogram plots were used to assess model assumptions of constant variance and independence of errors. Because all models showed evidence of heteroskedasticity and spatial autocorrelation of residuals, each model was also re-fitted with the exponential variance structure and exponential correlation structure and the model with the lowest Akaike Information Criterion (AIC) selected. No step-wise variable selection procedures were used, in order to avoid anticonservative p-values and inflated R^2^ given the large number of predictors. The resulting model was refitted using restricted maximum likelihood (REML) and its residuals assessed for homogeneity of variance, spatial independence and normality.
(DOCX)

S4 Appendix Comparison of soil variables from this study against data from John *et al*. (2007). SD = standard deviation. All variables except pH are in mg kg^-1^. Blanks represent unreported values. N = total mineral N, calculated as NO_3_^−^-N + NH_4_^+^-N. Note that statistics from Mudumalai were calculated using raw data while statistics from other sites were calculated using interpolated (kriged) data.
(DOCX)

## References

[1] Fine PVA, Daly DC, Cameron KM. The contribution of edaphic heterogeneiy to the evolution and diversity of Burseraceae trees in the western Amazon. Evolution. 2005;59: 1464–1478. 10.1111/j.0014-3820.2005.tb01796.x 16153032

[2] Toledo M, Peña-Claros M, Bongers F, Alarcón A, Balcázar J, Chuviña J, et al Distribution patterns of tropical woody species in response to climatic and edaphic gradients. J Ecol. 2012; 10.1111/j.1365-2745.2011.01890.x

[3] Clark DB, Clark DA, Read JM. Edaphic variation and the mesoscale distribution of tree species in a neotropical rain forest. J Ecol. 1998;86: 101–112. 10.1046/j.1365-2745.1998.00238.x

[4] Paoli GD, Curran LM, Zak DR. Soil nutrients and beta diversity in the Bornean Dipterocarpaceae: evidence for niche partitioning by tropical rain forest trees. J Ecol. 2006;94: 157–170. 10.1111/j.1365-2745.2005.01077.x

[5] John R, Dalling JW, Harms KE, Yavitt JB, Stallard RF, Mirabello M, et al Soil nutrients influence spatial distributions of tropical tree species. Proc Natl Acad Sci. 2007;104: 864–869. 10.1073/pnas.0604666104 17215353 PMC1783405

[6] Baldeck CA, Harms KE, Yavitt JB, John R, Turner BL, Valencia R, et al Soil resources and topography shape local tree community structure in tropical forests. Proc R Soc B Biol Sci. 2012;280 10.1098/rspb.2012.2532 PMC3574348 23256196

[7] Silvertown J. Plant coexistence and the niche. Trends Ecol Evol. 2004;19: 605–611. 16/j.tree.2004.09.003

[8] Lechowicz MJ, Bell G. The ecology and genetics of fitness in forest plants. II. Microspatial heterogeneity of the edaphic environment. J Ecol. 1991;79: 687–696. 10.2307/2260661

[9] Ettema CH, Wardle DA. Spatial soil ecology. Trends Ecol Evol. 2002;17: 177–183. 16/S0169-5347(02)02496-5

[10] Callaham MA, Rhoades CC, Heneghan L. A striking profile: soil ecological knowledge in restoration management and science. Restor Ecol. 2008;16: 604–607.

[11] Jenny H. Factors of soil formation. New York, London: McGraw-Hill Book Company; 1941.

[12] Schaetzl RJ, Anderson S. Soils: genesis and geomorphology. Cambridge University Press; 2005.

[13] Lavelle P, Blanchart E, Martin A, Martin S, Spain A. A hierarchical model for decomposition in terrestrial ecosystems: application to soils of the humid tropics. Biotropica. 1993;25: 130–150. 10.2307/2389178

[14] Mooney HA, Bullock SH, Medina E. Introduction In: Bullock SH, Mooney HA, Medina E, editors. Seasonally dry tropical forests. Cambridge University Press; 1995.

[15] Murphy PG, Lugo AE. Ecology of tropical dry forest. Annu Rev Ecol Syst. 1986;17: 67–88.

[16] Phillips JD, Marion DA. Biomechanical effects, lithological variations, and local pedodiversity in some forest soils of Arkansas. Geoderma. 2005;124: 73–89. 10.1016/j.geoderma.2004.04.004

[17] Raghubanshi AS. Effect of topography on selected soil properties and nitrogen mineralization in a dry tropical forest. Soil Biol Biochem. 1992;24: 145–150. 10.1016/0038-0717(92)90270-8

[18] Grogan J, Galvão J. Physiographic and floristic gradients across topography in transitional seasonally dry evergreen forests of southeast Pará, Brazil. Acta Amaz. 2006;36: 483–496. 10.1590/S0044-59672006000400009

[19] Cotler H, Ortega-Larrocea MP. Effects of land use on soil erosion in a tropical dry forest ecosystem, Chamela watershed, Mexico. CATENA. 2006;65: 107–117. 10.1016/j.catena.2005.11.004

[20] Roy S, Singh JS. Seasonal and spatial dynamics of plant-available N and P pools and N-mineralization in relation to fine roots in a dry tropical forest habitat. Soil Biol Biochem. 1995;27: 33–40. 10.1016/0038-0717(94)00138-Q

[21] Galicia L, López-Blanco J, Zarco-Arista AE, Filips V, Garcıía-Oliva F. The relationship between solar radiation interception and soil water content in a tropical deciduous forest in Mexico. CATENA. 1999;36: 153–164. 10.1016/S0341-8162(98)00121-0

[22] Singh JS, Kashyap AK. Variations in soil N-mineralization and nitrification in seasonally dry tropical forest and savanna ecosystems in Vindhyan region, India. Trop Ecol. 2007;48: 27–36.

[23] Binkley D. The influence of tree species on forest soils: processes and patterns. Spec Publ-Agron Soc N Z. 1995; 1–34.

[24] Binkley D, Giardina C. Why do tree species affect soils? The warp and woof of tree-soil interactions. Biogeochemistry. 1998;42: 89–106. 10.1023/A:1005948126251

[25] Rodríguez A, Durán J, Fernández-Palacios JM, Gallardo A. Spatial pattern and scale of soil N and P fractions under the influence of a leguminous shrub in a Pinus canariensis forest. Geoderma. 2009;151: 303–310. 10.1016/j.geoderma.2009.04.019

[26] Certini G. Effects of fire on properties of forest soils: a review. Oecologia. 2005;143: 1–10. 10.1007/s00442-004-1788-8 15688212

[27] Binkley D, Fisher R. Ecology and management of forest soils. John Wiley & Sons; 2012.

[28] Hirobe M, Tokuchi N, Wachrinrat C, Takeda H. Fire history influences on the spatial heterogeneity of soil nitrogen transformations in three adjacent stands in a dry tropical forest in Thailand. Plant Soil. 2003;249: 309–318. 10.1023/A:1022804326662

[29] McShea W, Davies S. Introduction: seasonally dry forests of tropical Asia In: McShea W, Davies S, Bhumpakphan N, editors. Th ecology and conservation of seasonally dry forests in Asia. Washington, DC: Smithsonian Institution Scholarly Press; 2011 pp. 59–74.

[30] Døckersmith IC, Giardina CP, Sanford RL Jr. Persistence of tree related patterns in soil nutrients following slash-and-burn disturbance in the tropics. Plant Soil. 1999;209: 137–157. 10.1023/A:1004503023973

[31] Giardina CP, Sanford RL, Døckersmith IC. Changes in soil phosphorus and nitrogen during slash-and-burn clearing of a dry tropical forest. Soil Sci Soc Am J. 2000;64: 399 10.2136/sssaj2000.641399x

[32] Soltis DE, Soltis PS, Morgan DR, Swensen SM, Mullin BC, Dowd JM, et al Chloroplast gene sequence data suggest a single origin of the predisposition for symbiotic nitrogen fixation in angiosperms. Proc Natl Acad Sci. 1995;92: 2647–2651. 10.1073/pnas.92.7.2647 7708699 PMC42275

[33] Sukumar R, Suresh HS, Dattaraja HS, Srinidhi S, Nath C. The dynamics of a tropical dry forest in India: climate, fire, elephants and the evolution of life-history strategies In: Burslem DFRP, Pinard MA, Hartley SE, editors. Biotic interactions in the tropics: Their role in the maintenance of species diversity. 2005 pp. 510–529.

[34] Drury SA, Holt RW. The tectonic framework of the South Indian craton: A reconnaissance involving LANDSAT imagery. Tectonophysics. 1980;65: T1–T15. 10.1016/0040-1951(80)90073-6

[35] Srikantappa C, Prakash Narasimha KN. Retrogression of charnockites in Moyar Shear Zone, Tamil Nadu, India. Mem—Geol Soc India. 1988;11: 117–24.

[36] Chardon D, Jayananda M, Chetty TRK, Peucat J-J. Precambrian continental strain and shear zone patterns: South Indian case. J Geophys Res Solid Earth. 2008;113: B08402 10.1029/2007JB005299

[37] Meißner B, Deters P, Srikantappa C, Köhler H. Geochronological evolution of the Moyar, Bhavani and Palghat shear zones of southern India: implications for east Gondwana correlations. Precambrian Res. 2002;114: 149–175. 10.1016/S0301-9268(01)00222-4

[38] Gunnell Y. The interaction between geological structure and global tectonics in multistoreyed landscape development: a denudation chronology of the South Indian shield. Basin Res. 1998;10: 281–310. 10.1046/j.1365-2117.1998.00072.x

[39] Braun J-J, Descloitres M, Riotte J, Fleury S, Barbiéro L, Boeglin J-L, et al Regolith mass balance inferred from combined mineralogical, geochemical and geophysical studies: Mule Hole gneissic watershed, South India. Geochim Cosmochim Acta. 2009;73: 935–961. 10.1016/j.gca.2008.11.013

[40] Violette A, Goddéris Y, Maréchal J-C, Riotte J, Oliva P, Kumar MSM, et al Modelling the chemical weathering fluxes at the watershed scale in the Tropics (Mule Hole, South India): Relative contribution of the smectite/kaolinite assemblage versus primary minerals. Chem Geol. 2010;277: 42–60. 10.1016/j.chemgeo.2010.07.009

[41] Violette A, Riotte J, Braun J-J, Oliva P, Marechal J-C, Sekhar M, et al Formation and preservation of pedogenic carbonates in South India, links with paleo-monsoon and pedological conditions: Clues from Sr isotopes, U–Th series and REEs. Geochim Cosmochim Acta. 2010;74: 7059–7085. 10.1016/j.gca.2010.09.006

[42] George M, Gupta G, Singh J. Forest soil vegetation survey report on Mudumalai Forest Division, Tamilnadu. For Soil–Vegetation Surv South Reg Coimbatore India. 1988;

[43] Kodandapani N, Cochrane MA, Sukumar R. A comparative analysis of spatial, temporal, and ecological characteristics of forest fires in seasonally dry tropical ecosystems in the Western Ghats, India. For Ecol Manag. 2008;256: 607–617. 10.1016/j.foreco.2008.05.006

[44] Sukumar R, Dattaraja HS, Suresh HS, Radhakrishnan J, Vasudeva R, Nirmala S, et al Long-term monitoring of vegetation in a tropical deciduous forest in Mudumalai, southern India. Curr Sci. 1992;62: 608–616.

[45] Condit R. Tropical forest census plots: methods and results from Barro Colorado Island, Panama and a comparison with other plots. Springer; 1998.

[46] Beven KJ, Kirkby MJ. A physically based, variable contributing area model of basin hydrology. Hydrol Sci Bull. 1979;24: 43–69. 10.1080/02626667909491834

[47] Fu P, Rich PM. Design and implementation of the Solar Analyst: an ArcView extension for modeling solar radiation at landscape scales. Proceedings of the 19th annual ESRI user conference, San Diego, USA. 1999.

[48] Gessler PE, Chadwick OA, Chamran F, Althouse L, Holmes K. Modeling soil-landscape and ecosystem properties using terrain attributes. Soil Sci Soc Am J. 2000;64: 2046 10.2136/sssaj2000.6462046x

[49] Moore ID, Gessler PE, Nielsen GA, Peterson GA. Soil attribute prediction using terrain analysis. Soil Sci Soc Am J. 1993;57: 443 10.2136/sssaj1993.03615995005700020026x

[50] Seibert J, Stendahl J, Sørensen R. Topographical influences on soil properties in boreal forests. Geoderma. 2007;141: 139–148. 10.1016/j.geoderma.2007.05.013

[51] R Core Team. R: A Language and Environment for Statistical Computing [Internet]. Vienna, Austria: R Foundation for Statistical Computing; 2013 Available: http://www.R-project.org/

[52] Fortin M-J, Jacquez GM. Randomization tests and spatially auto-correlated data. Bull Ecol Soc Am. 2000;81: 201–205.

[53] Samuel VO, Santosh M, Liu S, Wang W, Sajeev K. Neoarchean continental growth through arc magmatism in the Nilgiri Block, southern India. Precambrian Res. 2014;245: 146–173. 10.1016/j.precamres.2014.02.002

[54] Mengel K, Kirkby EA, Kosegarten H, Appel T. Principles of plant nutrition. Springer; 2001.

[55] Dutilleul P, Clifford P, Richardson S, Hemon D. Modifying the t test for assessing the correlation between two spatial processes. Biometrics. 1993;49: 305–314. 10.2307/2532625 2720048

[56] Benjamini Y, Hochberg Y. Controlling the false discovery rate: a practical and powerful approach to multiple testing. J R Stat Soc Ser B Methodol. 1995; 289–300.

[57] Barbiéro L, Parate HR, Descloitres M, Bost A, Furian S, Mohan Kumar MS, et al Using a structural approach to identify relationships between soil and erosion in a semi-humid forested area, South India. CATENA. 2007;70: 313–329. 10.1016/j.catena.2006.10.013

[58] Sharma A, Rajamani V. Weathering of gneissic rocks in the upper reaches of Cauvery river, south India: implications to neotectonics of the region. Chem Geol. 2000;166: 203–223. 10.1016/S0009-2541(99)00222-3

[59] Gunnell Y. Granite landforms of the Indian cratons In: Kale VS, editor. Landscapes and landforms of India. Dordrecht: Springer Netherlands; 2014 p. 195–. Available: http://link.springer.com/10.1007/978-94-017-8029-2

[60] Mehta P. Impact of climate on geochemical mobilization of elements during rock weathering in Kaveri river catchment [Internet]. PhD Thesis, Jawaharlal Nehru University. 2014. Available: http://hdl.handle.net/10603/29866

[61] Durand N, Gunnell Y, Curmi P, Ahmad SM. Pedogenic carbonates on Precambrian silicate rocks in South India: Origin and paleoclimatic significance. Quat Int. 2007;162–163: 35–49. 10.1016/j.quaint.2006.10.026

[62] Schaetzl R. Catenas and soils In: Shroder JF, Pope G, editors. Treatise on Geomorphology. San Diego, CA: Academic Press; 2013 pp. 145–158.

[63] Montaño NM, Sandoval-Pérez AL, Nava-Mendoza M, Sánchez-Yañez JM, García-Oliva F. Spatial and seasonal variation of soil culturable-bacterial functional groups in a Mexican tropical dry forest. Rev Biol Trop. 2013;61: 439–453. 23894994

[64] Schwertmann U. Solubility and dissolution of iron oxides In: Chen Y, Hadar Y, editors. Iron Nutrition and Interactions in Plants. Springer Netherlands; 1991 pp. 3–27. Available: http://link.springer.com/chapter/10.1007/978-94-011-3294-7_1

[65] Maréchal J-C, Varma MRR, Riotte J, Vouillamoz J-M, Kumar MSM, Ruiz L, et al Indirect and direct recharges in a tropical forested watershed: Mule Hole, India. J Hydrol. 2009;364: 272–284. 10.1016/j.jhydrol.2008.11.006

[66] Ruiz L, Varma MRR, Kumar MSM, Sekhar M, Maréchal J-C, Descloitres M, et al Water balance modelling in a tropical watershed under deciduous forest (Mule Hole, India): Regolith matric storage buffers the groundwater recharge process. J Hydrol. 2010;380: 460–472. 10.1016/j.jhydrol.2009.11.020

[67] Barbiero L, Kumar MSM, Violette A, Oliva P, Braun JJ, Kumar C, et al Ferrolysis induced soil transformation by natural drainage in Vertisols of sub-humid South India. Geoderma. 2010;156: 173–188. 10.1016/j.geoderma.2010.02.014

[68] Riotte J, Maréchal JC, Audry S, Kumar C, Bedimo Bedimo JP, Ruiz L, et al Vegetation impact on stream chemical fluxes: Mule Hole watershed (South India). Geochim Cosmochim Acta. 2014;145: 116–138. 10.1016/j.gca.2014.09.015

[69] Holloway JM, Dahlgren RA. Nitrogen in rock: Occurrences and biogeochemical implications. Glob Biogeochem Cycles. 2002;16: 1118 10.1029/2002GB001862

[70] Vitousek PM, Walker LR. Biological invasion by *Myrica faya* in Hawai’i: plant demography, nitrogen fixation, ecosystem effects. Ecol Monogr. 1989;59: 247–265. 10.2307/1942601

[71] Drechsel P, Zech W. Foliar nutrient levels of broad-leaved tropical trees: A tabular review. Plant Soil. 1991;131: 29–46.

[72] Townsend AR, Cleveland CC, Asner GP, Bustamante MMC. Controls over foliar N:P ratios in tropical rain forests. Ecology. 2007;88: 107–118. 10.1890/0012-9658(2007)88[107:COFNRI]2.0.CO;2 17489459

[73] Singh J, Raghubanshi A, Singh R, Srivastava S. Microbial biomass acts as a source of plant nutrients in dry tropical forest and savanna. Nature. 1989;338: 499–500.

[74] Roy S, Singh J. Consequences of habitat heterogeneity for availability of nutrients in a dry tropical forest. J Ecol. 1994; 503–509.

[75] Mohan Kumar B, Deepu JK. Litter production and decomposition dynamics in moist deciduous forests of the Western Ghats in Peninsular India. For Ecol Manag. 1992;50: 181–201. 10.1016/0378-1127(92)90335-7

[76] Garcia-Oliva F, Martinez Lugo R, Maass JM. Long-term net soil erosion as determined by 137Cs redistribution in an undisturbed and perturbed tropical deciduous forest ecosystem. Geoderma. 1995;68: 135–147. 10.1016/0016-7061(95)00030-R

[77] Roy S. Spatial variation of soil physico-chemical properties influenced by spatial and temporal variation of litter in a dry tropical forest floor. Oecologia Mont. 1996;5: 21–26.

[78] Campo J, Maass JM, de Pablo L. Weathering in a Mexican tropical dry forest. Agrociencia. 2001;35: 245–254.

[79] DeBano LF. The role of fire and soil heating on water repellency in wildland environments: a review. J Hydrol. 2000;231–232: 195–206. 10.1016/S0022-1694(00)00194-3

[80] Mondal N, Sukumar R. Fire and soil temperatures during controlled burns in seasonally dry tropical forests of southern India. Curr Sci. 2014;107: 1590–1594.

[81] Gunnell Y, Bourgeon G. Soils and climatic geomorphology on the Karnataka plateau, peninsular India. CATENA. 1997;29: 239–262. 10.1016/S0341-8162(96)00070-7

[82] Riotte J, Ruiz L, Audry S, Sekhar M, Mohan Kumar MS, Siva Soumya B, et al Impact of vegetation and decennial rainfall fluctuations on the weathering fluxes exported from a dry tropical forest (Mule Hole). Procedia Earth Planet Sci. 2014;10: 34–37. 10.1016/j.proeps.2014.08.007

[83] Chadwick OA, Chorover J. The chemistry of pedogenic thresholds. Geoderma. 2001;100: 321–353. 10.1016/S0016-7061(01)00027-1

[84] Maher K. The role of fluid residence time and topographic scales in determining chemical fluxes from landscapes. Earth Planet Sci Lett. 2011;312: 48–58. 10.1016/j.epsl.2011.09.040

[85] Braun J-J, Marechal J-C, Riotte J, Boeglin J-L, Bedimo Bedimo J-P, Ndam Ngoupayou JR, et al Elemental weathering fluxes and saprolite production rate in a Central African lateritic terrain (Nsimi, South Cameroon). Geochim Cosmochim Acta. 2012;99: 243–270. 10.1016/j.gca.2012.09.024

[86] Campo J, Jaramillo VJ, Maass JM. Pulses of soil phosphorus availability in a Mexican tropical dry forest: effects of seasonality and level of wetting. Oecologia. 1998;115: 167–172. 10.1007/s004420050504 28308448

[87] Porder S, Chadwick OA. Climate and soil-age constraints on nutrient uplift and retention by plants. Ecology. 2009;90: 623–636. 19341134 10.1890/07-1739.1

[88] Vitousek P, Chadwick O, Matson P, Allison S, Derry L, Kettley L, et al Erosion and the rejuvenation of weathering-derived nutrient supply in an old tropical landscape. Ecosystems. 2003;6: 762–772. 10.1007/s10021-003-0199-8

[89] Bern CR, Townsend AR, Farmer GL. Unexpected dominance of parent-material strontium in a tropical forest on highly weathered soils. Ecology. 2005;86: 626–632. 10.1890/03-0766

[90] Porder S, Clark DA, Vitousek PM. Persistence of rock-derived nutrients in the wet tropical forests of La Selva, Costa Rica. Ecology. 2006;87: 594–602. 16602289 10.1890/05-0394

[91] Khomo L, Hartshorn AS, Rogers KH, Chadwick OA. Impact of rainfall and topography on the distribution of clays and major cations in granitic catenas of southern Africa. CATENA. 2011;87: 119–128. 10.1016/j.catena.2011.05.017

