# Supplementary material for: Controls of Soil Spatial Variability in a Dry Tropical Forest
Source: PLoS One. 2016 Apr 21;11(4):e0153212. doi: 10.1371/journal.pone.0153212 (PMC4839752; doi:10.1371/journal.pone.0153212)
Supplement: S1 Appendix — (DOCX) [file pone.0153212.s001.docx]

| No. | Soil variable | Observed value | Reason for removal of observation |
| --- | --- | --- | --- |
| 1 | P | 259.11 mg kg^-1^ | Nearly double the next biggest value (131.38 mg kg^-1^) and spatially surrounded by moderate values |
| 2 | K | 2014.94 mg kg^-1^ | Nearly 3 times greater than the next biggest value (791.54 mg kg^-1^) and spatially surrounded by moderate values |
| 3 | Zn | 10.12 mg kg^-1^ | Nearly double the next biggest value (5.86 mg kg^-1^) and spatially surrounded by moderate values |
| 4 | NO_3_^-^-N | 23.08 mg kg^-1^ | High value spatially surrounded by very low values |
| 5 | NO_3_^-^-N | 36.66 mg kg^-1^ | High value spatially surrounded by low values |
| 6 | NO_3_^-^-N | 18.82 mg kg^-1^ | High value spatially surrounded by low values |
| 7 | NH_4_^+^-N | 58.62 mg kg^-1^ | Nearly double the next biggest value (31.23 mg kg^-1^) and spatially surrounded by moderate values |
