# Supplementary figures and images for: Controls of Soil Spatial Variability in a Dry Tropical Forest
Source: PLoS One. 2016 Apr 21;11(4):e0153212. doi: 10.1371/journal.pone.0153212 (PMC4839752; doi:10.1371/journal.pone.0153212)

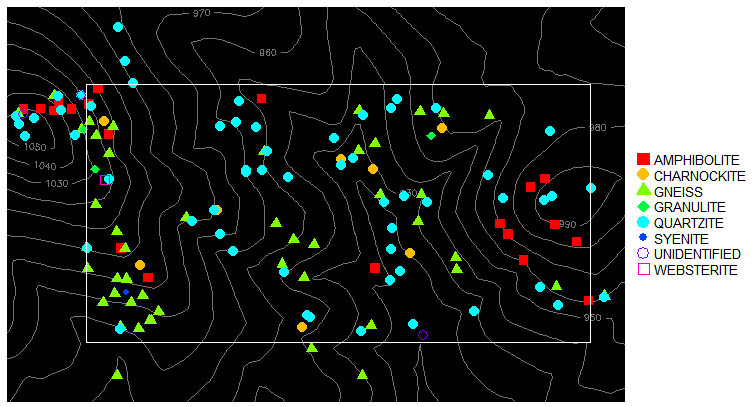

Supplement: S2 Appendix — (TIFF) [file pone.0153212.s002.tiff]
