# Supplementary material for: Controls of Soil Spatial Variability in a Dry Tropical Forest
Source: PLoS One. 2016 Apr 21;11(4):e0153212. doi: 10.1371/journal.pone.0153212 (PMC4839752; doi:10.1371/journal.pone.0153212)
Supplement: S3 Appendix — No predictors were significant for P and Fe. elev = elevation; slope = slope inclination; east = slope aspect (“eastness”); north = slope aspect (“northness”); curv = curvature; insol = insolation; twi = topographic wetness index; ba.fabaceae = basal area of woody Fabaceae species; texture = soil texture class; fire = fire frequency (times burnt during 1988–2004); x, y = distances in west-east and south-north directions, respectively; TC = total carbon. Quadratic term of a given variable var is shown as var^2. All response variables and continuous predictors except fire were standardized as described in the text; fire was centered but not scaled. Quadratic and first-order interaction terms were included where deemed necessary, based on scatter plots and prior expectations. Predictors were tested for multicollinearity based on variance inflation factors (VIF) and correlations between coefficient estimates. Because TWI and insolation were correlated with the remaining topographic variables, separate regressions were built using two subsets of mutually uncorrelated predictors. Residual plots and empirical variogram plots were used to assess model assumptions of constant variance and independence of errors. Because all models showed evidence of heteroskedasticity and spatial autocorrelation of residuals, each model was also re-fitted with the exponential variance structure and exponential correlation structure and the model with the lowest Akaike Information Criterion (AIC) selected. No step-wise variable selection procedures were used, in order to avoid anticonservative p-values and inflated R2 given the large number of predictors. The resulting model was refitted using restricted maximum likelihood (REML) and its residuals assessed for homogeneity of variance, spatial independence and normality. (DOCX) [file pone.0153212.s003.docx]

B = 0.51*elev + 0.11*slope + 0.35*pH

Mg = 0.14*slope - 0.33*texture Sandy Clay Loam + 0.1*pH + 0.21*elev*slope - 0.12*curv*north + 0.21*elev - 0.02*curv - 0.03*north

Al = -0.26*texture Sandy Clay Loam - 0.44*pH + 0.11*twi*insol + 0.1*twi - 0.08*insol

K = -0.26*x + 0.23*pH - 0.14*elev*curv + 0.11*curv*east - 0.15*curv*north + 0.04*elev - 0.06*curv - 0.05*east - 0.08*north

Ca = 0.35*elev + 0.12*slope - 0.3*texture Sandy Clay Loam + 0.24*pH + 0.16*elev*slope

Mn = 0.54*elev + 0.17*slope + 0.07*slope^2 - 0.2*texture Sandy Clay Loam + 0.22*x + 0.11*pH + 0.12*elev*east + 0.08*east

Cu = 0.52*elev + 0.08*slope^2 - 0.32*texture Sandy Clay Loam + 0.24*x + 0.09*slope

Zn = -0.24*twi + 0.15*pH

NO_3_^-^-N = -0.23*x + 0.15*pH

NH_4_^+^-N = -0.16*fire - 0.41*x + 0.1*pH

pH = 0.28*elev - 0.35*elev^2 + 0.19*slope*curv + 0.04*slope - 0.11*curv

TC =-0.37*twi - 0.6*texture Sandy Clay Loam

moisture = -0.18*fire
