# Supplementary material for: Controls of Soil Spatial Variability in a Dry Tropical Forest
Source: PLoS One. 2016 Apr 21;11(4):e0153212. doi: 10.1371/journal.pone.0153212 (PMC4839752; doi:10.1371/journal.pone.0153212)
Supplement: S4 Appendix — SD = standard deviation. All variables except pH are in mg kg-1. Blanks represent unreported values. N = total mineral N, calculated as NO3−-N + NH4+-N. Note that statistics from Mudumalai were calculated using raw data while statistics from other sites were calculated using interpolated (kriged) data. (DOCX) [file pone.0153212.s004.docx]

|  | Mudumalai,  India | | BCI,  Panama | | La Planada,  Colombia | | Yasuni,  Ecuador | |
| --- | --- | --- | --- | --- | --- | --- | --- | --- |
|  | Mean | SD | Mean | SD | Mean | SD | Mean | SD |
| N | 11.4 | 4 | 25.92 | 7.96 | 22.71 | 7.11 | 10.84 | 4.08 |
| B | 0.7 | 0.5 | 0.944 | 0.536 |  | 63.78 |  | 275.4 |
| Mg | 205.7 | 101.2 | 298.9 | 128 | 26.1 | 5.5 | 112.5 | 95 |
| Al | 137.9 | 64.1 | 1013.8 | 233.3 | 3732.4 | 234.3 | 1796.7 | 278.8 |
| P | 20.2 | 27.4 | 2.9 | 1.62 | 20.2 | 0.48 | 6.34 | 0.55 |
| K | 230.1 | 120.6 | 171.8 | 74.7 | 62.5 | 7.5 | 99.8 | 65.2 |
| Ca | 2509.4 | 971.9 | 1732.5 | 743.2 | 168.6 | 0.43 | 409.9 | 0.49 |
| Mn | 84.3 | 53.6 | 370.7 | 155.6 | 3.9 | 4.2 | 139.8 | 3.01 |
| Fe | 215.6 | 62.4 | 178.5 | 46.2 | 562.2 | 18 | 284.1 | 112 |
| Cu | 2.5 | 1.5 | 8.08 | 2.04 | 2.2 | 219.1 | 1.06 | 82.4 |
| Zn | 2.3 | 1.0 | 5.66 | 4.14 | 1.28 | 0.29 | 1.44 | 0.31 |
| pH | 6.7 | 0.4 | 5.66 | 0.34 | 4.46 |  | 4.63 |  |
